# Cortical morphometry of the five-factor model of personality: findings from the Human Connectome Project full sample

**DOI:** 10.1093/scan/nsz017

**Published:** 2019-03-08

**Authors:** Max M Owens, Courtland S Hyatt, Joshua C Gray, Nathan T Carter, James MacKillop, Joshua D Miller, Lawrence H Sweet

**Affiliations:** 1 Department of Psychology, University of Georgia, Athens, GA, USA; 2 Department of Medical and Clinical Psychology, Uniformed Services University, Bethesda, MD, USA; 3 Peter Boris Centre for Addiction Research, St. Joseph’s Healthcare Hamilton/McMaster University, West 5th Street, Hamilton, ON, Canada

**Keywords:** personality, MRI, five-factor model, morphometry, traits

## Abstract

This study is a replication of an existing large study (*N* = 507) on the surface-based morphometric correlates of five-factor model (FFM) personality traits. The same methods were used as the original study in another large sample drawn from the same population (*N* = 597) with results then being aggregated from both samples (*N* = 1104), providing the largest investigation into the neuroanatomical correlates of FFM personality traits to date. Clusters of association between brain morphometry and each FFM trait are reported. For neuroticism, agreeableness, openness and conscientiousness clusters of association were found in the dorsolateral prefrontal cortex for at least one morphometric index. Morphometry in various other regions was also associated with each personality trait. While some regions found in the original study were confirmed in the replication and full samples, others were not, highlighting the importance of replicating even high-quality, well-powered studies. Effect sizes were very similar in the replication and whole samples as those found in the original study. As a whole, the current results provide the strongest evidence to date on the neuroanatomical correlates of personality and highlights challenges in using this approach to understanding the neural correlates of personality.

## Introduction

The five-factor model (FFM) of personality has been a guiding framework for understanding how personality traits are related to a range of important biological, psychological and social phenomena (McCrae and Costa, 1997). Traits include (i) neuroticism, which is associated with negative emotional reactivity; (ii) extraversion, which is associated with a drive for affiliation and dominance; (iii) openness, which is associated with fantasy, interest in novelty and intellect; (iv) agreeableness, which is associated with nurturance, kindness and social harmony; and (v) conscientiousness, which is associated with orderliness and impulse control (John and Srivastava, 1999). Due in part to technological advancements, as well as the promulgation of major research initiatives that encourage investigations into the biological foundations of psychological phenomena (e.g. Research Domain Criteria; Insel *et al.*, 2010; Cuthbert and Insel, 2013), research on the neural bases of personality has burgeoned in the past decade (e.g. Allen and DeYoung, 2015), which is particularly relevant given personality’s links to psychopathology (Kotov *et al.*, 2017) and a wide array of important outcomes (Ozer and Benet-Martinez, 2006; Soto, submitted for publication). Historically, there are structural models of personality that are explicitly posited to capture specific physiological processes (e.g. Cloninger’s temperament and character model, Cloninger *et al.*, 1993; Eysenck’s PEN model, Eysenck and Eysenck, 1970), but the FFM has its roots in the lexical hypothesis, which proposes that the most socially relevant information will be encoded most robustly in language (see John and Srivastava, 1999 for a review). Although the FFM was not initially constructed to capture specific physiological processes, it is amenable to predictions about which regions of the brain are relevant to particular traits (e.g. DeYoung *et al.*, 2010). For example, extraversion is partially comprised of the facet dominance, and thus, it could be hypothesized that it is associated with regions of the brain related to approach orientation (e.g. nucleus accumbens, medial prefrontal cortex, cingulate gyrus, amygdala; Machado and Cantilino, 2016). Although several studies report structural magnetic resonance imaging (MRI) results indicating associations of gray matter volume in these regions with extraversion, the direction of findings have been inconsistent across studies (Omura *et al.*, 2005; Cremers *et al.*, 2017).

In the literature review for their recent study, Riccelli *et al.*, (2017) suggest that findings across all FFM traits are inconsistent to date (e.g. Coutinho *et al.,*^2013^; DeYoung *et al.*, 2010). There are several methodological considerations that may contribute to this heterogeneity, including low statistical power related to small sample sizes (Button *et al.*, 2013) and wide variation in age range across studies (e.g. Fjell and Walhovd, 2010). Furthermore, most of the extant work on the FFM and neuroanatomy has used voxel-based morphometry to analyze the gray matter variance associated with FFM traits. Because of voxel-based morphometry’s reliance on the measured intensity of voxels to assign a probability that each voxel represents gray or white matter, its index of gray matter volume can be difficult to interpret. Voxel-based morphometry is also unable to assess specific features of cortical gray matter, such as cortical thickness and cortical surface area (Hutton *et al.*, 2009).

Noting these limitations, Riccelli *et al*. (2017) employed a surface-based morphometry approach (SBM; i.e. quantification of cortical morphometry) to investigate the neuroanatomical correlates of FFM traits using a large sample comprising approximately half (*N* = 507) of the participants in the Human Connectome Project (HCP). Results suggest each trait was characterized by unique relations to SBM metrics. In brief, neuroticism was associated with a thicker cortical ribbon and a smaller surface area in prefrontal and temporal regions. Extraversion was linked to a thicker cortical ribbon in the precuneus, reduced gray matter volume in the superior temporal gyrus (STG) and entorhinal cortex and greater cortical gyrification in the fusiform gyrus. Openness was correlated with thinner cortices, but greater area throughout the parietal, temporal and frontal lobes. Agreeableness was associated with lower cortical thickness, area and volume in the frontal and temporal regions. Finally, conscientiousness was correlated with thicker prefrontal cortex and smaller surface area and gyrification in the middle/inferior temporal and lateral occipital gyrus. These authors concluded that there are numerous areas and metrics of the brain associated with personality traits, with the prefrontal cortex seeming to be the most important.

### The current study

Since the publication of this article by Riccelli *et al*. (2017), the size of the available HCP data set has more than doubled. In the current study, we first aimed to independently replicate the findings of Riccelli *et al*. (2017) using the 597 participants from the HCP data set who were not included in their analyses. Second, we aimed to aggregate the results of the two studies across the full sample of 1104. In doing so, the current study provides the most well-powered investigation into the neuroanatomical correlates of FFM traits to date.

In addition to attempting to replicate the findings of (Riccelli *et al.*, 2017) in a larger sample, the current study also reports a secondary alternative analytic approach in which the relations between an FFM trait and SBM indices were examined while not controlling for the other four FFM traits. This was done due to documented construct validity concerns about meaningfully interpreting partialed personality variables, in that relations can be attenuated, or new and/or stronger relations can be discovered due to statistical suppressor effects (i.e. ‘perils of partialing’; Lynam *et al.*, 2006; Sleep *et al.*, 2017). In this analysis, we reanalyzed the full sample results without using other FFM traits as covariates.

## Methods

### Participants

Structural MRI and self-reported personality data were collected from 1104 participants at Washington University as part of HCP between August 2012 and October 2015 and released in full on 1 March 2017. Demographic information about the full sample can be found in Table 1, alongside information about participants in the replication sample (*N* = 597) that were not included in the original sample (*N* = 507; Riccelli *et al.*, 2017). There were statistically significant differences between the two samples in age (29.2 years *vs* 28.5 years; *t* = 3.27, *P* = 0.001; Cohen’s d = 0.19), race (22% *vs* 9.0% African-American; 2% *vs* 9% Asian-American; *χ^2^* = 67.32, *P* < 0.001) and ethnicity (10% *vs* 8% Hispanic or Latino; *χ^2^* = 10.31, *P* < 0.006). No other demographic factors differed between samples. Additionally, there were no significant differences in any FFM traits between the samples (all *P* > 0.1, Cohen’s *d* < 0.1)*.* All participants provided informed consent (Van Essen *et al.*, 2013a). Exclusion criteria included a history of severe psychiatric (e.g. schizophrenia), neurological (e.g. traumatic brain injury) or medical disorders (e.g. cardiovascular disease, Mendelian genetic disease). Additionally, participants did not have any MRI contraindications such as unsafe metal implants or claustrophobia (for full details of inclusion and exclusion criteria, see Van Essen *et al.*, 2013b).

**Table 1:** Demographic characteristics of the original sample, the replication sample and the combined samples. ^*^Indicates statistically significant differences between original and partial sample (*P* < 0.05)

	**Original sample (*N* = 507)**	**Replication sample (*N* = 597)**	**Full Sample (*N* = 1104)**
Sex			
Male	40.6%	50.1%	45.7%
Female	59.4%	49.9%	54.3%
Age^*^	29.2 (3.5)	28.5 (3.8)	28.8 (3.7)
Race^*^			
White or Caucasian	72.6%	76.5%	74.7%
Black or African American	22.3%	9.0%	15.1%
Asian American	1.8%	9.0%	5.7%
Native American	0.0%	0.3%	0.2%
More than one race	1.2%	3.7%	2.5%
Not sure or unknown	2.2%	1.3%	1.7%
Ethnicity^*^			
Hispanic or Latino	10.1%	7.6%	8.5%
Not Hispanic or Latino	89.7%	90.8%	90.3%
Not sure or unknown	0.2%	2.0%	1.2%
Income			
$1000–$9999/year	7.9%	6.4%	7.1%
$10 000–$19 999/year	7.7%	8.0%	7.9%
$20 000–$29 999/year	13.6%	11.4%	12.5%
$30 000–$39 999/year	10.8%	12.9%	12.0%
$40 000–$49 999/year	10.8%	9.7%	10.3%
$50 000–$74 999/year	18.7%	22.9%	21.1%
$75 000–$99 999/year	15.4%	11.6%	13.4%
$100 000–$149 999/year	14.4%	16.4%	15.6%
Years of education	14.8	15.0 (1.7)	14.92 (1.80)

Note: Demographic information is presented as Mean (standard deviation) or as a percentage.

### Materials and procedures

#### NEO-FFI

The NEO-FFI is a 60-item self-report measure of FFM personality traits (Costa and McCrae, 1992). Cronbach’s α ranged 0.76 to 0.85 in the replication sample and 0.75 to 0.84 in the full sample. In the full sample, intercorrelations ranged from *r* = −0.40 (neuroticism-conscientiousness) to 0.28 (extraversion-agreeableness). Of note, an error was detected in the calculation of agreeableness in the HCP download (see https://www.mail-archive.com/hcp-users@humanconnectome.org/msg06006.html for the correspondence regarding the error). We corrected this error by recalculating the agreeableness score.

#### MRI data acquisition

High-resolution T1-weighted structural images were acquired on a 3T Siemens Skyra scanner (Siemens AG, Erlanger, Germany) with a 32-channel head coil at a resolution of 0.7 mm^3^ isotropic (Field of View  = 224 × 240, matrix = 320 × 320, 256 sagittal slices; repetition time (TR) = 2400 ms and Echo Time (TE) = 2.14 ms). The quality checking procedure completed to ensure all scans were of high quality is documented in Marcus *et al*. 2013.

#### Data processing and analysis

Data were reconstructed and preprocessed using the Freesurfer pipeline (Dale *et al.*, 1999; Fischl *et al.*, 1999; 2004) in FreeSurfer Image Analysis Suite version 5.3 (http://surfer.nmr.mgh.harvard.edu; Fischl, 2012). In this process, Freesurfer models boundaries between the cortical white matter, gray matter and pial surfaces in order to create a two-dimensional representation of the cortical surface. Steps in this process include removing non-brain tissue, correcting intensity non-uniformity, identifying the boundaries of gray and white matter, tessellating the gray–white boundary, inflating and flattening the cortical surface, transforming it into a spherical space and automatically parcellating the cortex. See Glasser *et al.* (2013) and Van Essen *et al.* (2013b) for more details of the specific preprocessing pipeline used in the HCP. Four indices were investigated: cortical thickness, cortical surface area, gray matter volume and local gyrification index (LGI). Cortical thickness represents the distance from a vertex on the pial surface (outer surface) to the corresponding vertex on the gray–white matter boundary (inner surface). Cortical surface area represents the average area in each of the surrounding triangles to a given vertex. Gray matter volume is derived by multiplying cortical thickness and surface area values at a given vertex. LGI is a metric of how much of the cortex is buried in the folds of the sulci (relative to the amount on the surface) and was calculated at each vertex across the cortical surface.

Freesurfer version 5.3 (Fischl, 2012) was also used to conduct group-level vertex-wise cortical surface analysis to assess the relationship of brain structure with FFM traits at each vertex across the brain in an atheoretical approach. This approach is described on the Freesurfer Wiki (https://surfer.nmr.mgh.harvard.edu/fswiki/FsTutorial/GroupAnalysis) and was utilized in the study being replicated in the current analysis (Riccelli *et al.*, 2017). In this procedure, the outputs of reconstruction and preprocessing described above were resampled into a common space, combined into a single file and spatially smoothed. Then general linear modeling was conducted to determine associations of each FFM trait with the morphometric index of interest (e.g. cortical thickness, cortical surface area) at each vertex across the cortex.

Vertex-wise general linear modeling was completed for each morphometric index and FFM trait to determine cluster of vertices for which there was a significant association between that index and FFM trait. Clusterwise correction for multiple comparisons was then completed using Monte Carlo simulations (Hagler *et al.*, 2006). In this process, data were tested against a null distribution of maximum cluster size with an initial cluster-forming threshold of *P* < 0.05 as was done in Riccelli *et al.,* (2017). This yielded clusters corrected for multiple comparisons based on the total number of comparisons on the surface. Using this method, regions in which gray matter volume, cortical surface area, cortical thickness and LGI were associated with FFM traits at a cluster-corrected *P*-value of <0.05 were determined. Additionally, to ensure no important but smaller clusters of interest were omitted due to cluster size restriction, full-sample analyses were repeated using an initial cluster-forming threshold of *P* < 0.001 and are reported in Supplementary Materials. For further explanation of the interpretation of vertex-wise SBM, see Winkler *et al.*, 2012.

Consistent with the work of Riccelli *et al*. (2017), in order to correct for their confounding effects, analyses were conducted with total intracranial volume, age, sex and the other four FFM traits as covariates. In secondary analyses, we also completed analyses in the full sample while *not* controlling for the non-target FFM traits, the results of which are reported in Supplementary Table 2. Additionally, effect sizes were calculated for all clusters identified in vertex-wise analyses using the method indicated by Riccelli *et al*. (2017) and documented in the Freesurfer archives (https://www.mail-archive.com/freesurfer@nmr.mgh.harvard.edu/msg52144.html and https://www.mail-archive.com/freesurfer@nmr.mgh.harvard.edu/msg57316.html). These effect sizes represent the contrast to noise ratio and were calculated by multiplying the unstandardized regression coefficient by the contrast and dividing by the residual standard deviation of that vertex (i.e. cluster mean of $\frac{C\ast b}{SD_{res}}$ at each vertex). This creates an effect size metric ranging from −1 to 1 with 1 and −1 denoting perfect association with no noise (i.e. perfect positive and negative association) and 0 denoting no relationship considering the error present (https://www.mail-archive.com/freesurfer@nmr.mgh.harvard.edu/msg46806.html). This approach was used in order to conform most closely to the analytic approach of the study being replicated (Riccelli *et al*., 2017). See Supplementary Materials 1 for more information about the calculation and meaning of contrast to noise ratio.

## Results

### Replication sample analyses

Results of the replication sample (*N* = 597) analyses are reported in Supplementary Table 1. Effect sizes of the clusters found were generally similar to those identified in Riccelli *et al.* (2017) (between 0.01 and 0.02).

#### Neuroticism

In the replication sample (*N* = 597; see Supplementary Table 1), significant positive associations were found between neuroticism and thickness in a cluster in the left dorsolateral prefrontal cortex (DLPFC), ventrolateral prefrontal cortex (VLPFC), dorsomedial prefrontal cortex (DMPFC) and precentral gyrus; in a cluster in the left precentral gyrus, postcentral gyrus and inferior parietal lobule (IPL); in a cluster in the right DLPFC and DMPFC; and in a cluster in the right DLPFC. For surface area, clusters of negative association were found in the left DLPFC, left cuneus, left inferior temporal cortex, right DLPFC and DMPFC and right cuneus. The only cluster found for volume was a cluster of negative association in the left cuneus. For LGI, clusters of negative association were found in the left VLPFC, ventromedial prefrontal cortex (VMPFC), fusiform/lingual gyrus and IPL, as well as in the right temporoparietal junction/insula, DLPFC, VLPFC/lateral orbitofrontal cortex and precentral gyrus.

#### Extraversion

In the replication sample, a cluster of positive correlation was found between extraversion and volume in the right precentral gyrus. For LGI, clusters of positive association were found in the left IPL/lateral occipital cortex and the right IPL.

#### Openness

In the replication sample, clusters of negative association were found with thickness in the left DLPFC/VLPFC/DMPFC/precentral gyrus, left SPL, left IPL and right DLPFC/VLPFC/DMPFC. For surface area and volume, clusters of positive association were found in the left inferior temporal cortex and the right lateral occipital cortex. For LGI, clusters of positive association were found in the left VLPFC/lateral orbitofrontal and the right temporoparietal junction.

#### Agreeableness

In the replication sample, clusters of positive association were found between agreeableness and LGI in the left DLPFC, left precentral/postcentral gyrus and left DMPFC, as well as a cluster in the right temporoparietal junction, IPL and SPL, postcentral gyrus and lateral occipital cortex. Additionally, clusters of negative association were found between agreeableness and LGI in the left lingual gyrus and right parahippocampal gyrus.

#### Conscientiousness

In the replication sample, clusters of positive association were with thickness in the left and right DLPFC and clusters of negative association with area in the left and right DLPFC. Additionally, clusters of negative association were found with LGI in the left DLPFC/DMPFC, precentral/postcentral gyrus and postcentral gyrus/temporoparietal junction, as well as in the right temporoparietal junction and DLPFC/DMPFC.

### Full sample analyses

Results of the full sample (*N* = 1104) analyses are reported in Table 2. Effect sizes of the clusters found were generally similar to those identified in the study by Riccelli *et al.* (2017) (between 0.01 and 0.02). Table 3 displays the overlap of findings from the original sample, the replication sample and the full sample with clusters grouped by hemisphere, lobe and lateral/medial surface. Additionally, the results of the secondary analysis in the full sample which the other FFM traits were not included as covariates are reported in Supplementary Table 2 with areas of difference from the primary analyses indicated in the text below. In these analyses, effect sizes were slightly smaller than those controlling for the other four FFM traits. Furthermore, supplemental analyses in which an initial cluster forming threshold of *P* < 0.001 was used were generally similar to the primary analyses, but with clusters that were smaller, fewer in number and had slightly larger effect sizes. These analyses are reported in Supplementary Table 3.

**Table 2:** Significant clusters of brain by FFM trait correlation from the full sample (*N* = 1104) using other four FFM traits as covariates. Age, sex and total intracranial volume were also modeled as covariates. Max = maximum *P*-value in cluster, -log10 transformed; size = cluster size in mm^2^; X, Y, Z coordinates in MNI space; CWP = clusterwise probability; ES = effect size. DLPFC = dorsolateral prefrontal cortex; DMPFC = dorsomedial prefrontal cortex; VLPFC = ventrolateral prefrontal cortex; TPJ = temporal parietal junction

**Trait**	**Metric**	**Region**	**Max**	**Size**	**X**	**Y**	**Z**	**CWP**	**ES**
**N**	LH thickness	DLPFC/VLPFC/DMPFC	5.09	9209	−36	11	54	<0.0001	0.014
		Postcentral/inferior parietal	3.94	2877	−54	−7	19	<0.0001	0.013
	RH thickness	DLPFC/VLPFC/DMPFC	5.83	8130	15	39	42	<0.0001	0.014
		ITG	3.83	1347	58	−5	−24	0.003	0.013
		Lingual gyrus	2.42	1131	14	−78	−7	0.01	0.011
	LH area	Insula/superior temporal	−4.91	2043	−37	−25	−2	0.002	−0.014
		Lateral occipital	−4.79	2428	−41	−8	−38	<0.0001	−0.015
		Paracentral/DLPFC	−4.76	4897	−6	−18	64	<0.0001	−0.013
		Cuneus	−3.74	4866	−4	−88	12	<0.0001	−0.013
	RH area	DLPFC/DMPFC	−5.45	4918	9	47	43	<0.0001	−0.015
		ITG	−4.57	2780	38	9	−31	<0.0001	−0.013
		IPL	−3.55	1682	53	−47	25	0.01	−0.012
		Cuneus	−2.98	2588	8	−87	31	<0.0001	−0.012
	LH volume	Insula/superior temporal	−4.87	1502	−40	−26	−2	<0.0001	−0.013
		Cuneus	−3.49	1985	−21	−97	15	<0.0001	−0.012
		Lateral occipital	−2.83	1033	−34	−85	−1	0.02	−0.012
	RH volume	Cuneus	−3.06	1061	11	−91	14	0.02	−0.011
		DLPFC	−2.76	1240	16	48	31	0.008	−0.012
	LH LGI	DLPFC	−4.94	1070	−28	9	52	0.0002	−0.012
		Fusiform	−4.69	7513	−28	−57	−16	0.0002	−0.014
		DLPFC	−4.1	5937	−37	52	4	0.0002	−0.012
		Superior/inferior parietal	−3.53	6393	−30	−48	35	0.0002	−0.012
		Precentral gyrus/insula	−3.51	5194	−47	−8	26	0.0002	−0.011
		Paracentral	−2.37	718	−17	−25	38	0.004	−0.011
	RH LGI	Superior/inferior parietal	−4.89	8142	54	−24	23	0.0002	−0.013
		DLPFC	−3.63	3169	24	45	20	0.0002	−0.012
		Precentral gyrus/insula	−3.1	687	27	−21	65	0.0056	−0.011
		Medial orbitofrontal	−2.99	1142	24	17	−21	0.0002	−0.012
		Lateral occipital	−2.9	1382	31	−88	0	0.0002	−0.012
		Lingual gyrus	−2.79	799	22	−74	−9	0.0012	−0.011
		Superior/inferior parietal	−2.62	1739	29	−44	58	0.0002	−0.011
**E**	LH thickness	Inferior parietal	4.31	1862	−35	−73	45	<0.0001	0.015
		DMPFC/paracentral	2.95	1837	−4	−35	63	<0.0001	0.013
	RH volume	Precentral	3.42	1457	10	24	57	0.002	0.014
	LH LGI	Inferior parietal	−3.35	1890	−35	−88	11	0.0002	−0.013
	RH LGI	Fusiform gyrus	2.98	1369	33	−46	−19	0.0002	0.013
**O**	LH thickness	Superior parietal	−4.6	2007	−33	−55	59	<0.0001	−0.013
		DLPFC/VLPFC	−4.42	4416	−15	57	17	<0.0001	−0.013
	RH thickness	DLPFC/VLPFC	−3.66	3119	24	41	23	0	−0.013
	LH area	Inferior temporal	2.84	1312	−48	−12	−33	0.04	0.012
	LH volume	Inferior temporal	2.5	977	−36	−3	−42	0.03	0.012
	RH volume	Insula	4.49	1106	36	4	1	0.02	0.015
	LH LGI	Lateral/medial Orbitofrontal	4.6	2173	−22	43	−13	0.0002	0.013
		Inferior temporal	3.96	774	−49	−14	−37	0.0022	0.014
		TPL/lateral occipital	3.94	5015	-44	−40	−21	0.0002	0.013
	RH LGI	Lateral occipital	2.34	723	47	−68	−12	0.0042	0.011
**A**	LH thickness	DLPFC	−3.05	942	−22	22	56	0.004	−0.014
	RH thickness	Lingual gyrus	4.13	1342	20	−62	−8	0.003	0.014
	RH volume	DLPFC	3.45	941	39	7	46	0.05	0.016
	LH LGI	Lingual gyrus	−2.7	989	−12	−83	−13	0.0002	-0.014
	RH LGI	TPJ	3.59	3729	44	−52	25	0.0002	0.015

**Table 2:** (continued)

**Trait**	**Metric**	**Region**	**Max**	**Size**	**X**	**Y**	**Z**	**CWP**	**ES**
**C**	LH thickness	DLPFC/VLPFC	4.32	2475	−45	26	30	<0.0001	0.016
	RH thickness	DLPFC/VLPFC	4.85	2306	36	17	35	<0.0001	0.015
	LH area	Middle temporal gyrus	−3.25	1777	−63	-31	−13	0.01	−0.014
		DLPFC	−2.58	1645	−23	42	20	0.01	−0.014
	RH Area	DLPFC/DMPFC	−3.1	1705	16	40	42	0.01	−0.014
	LH LGI	Precentral gyrus	−4.1	3294	−51	−5	26	0.0002	−0.014
		DLPFC/DMPFC	−3.95	4316	−16	58	14	0.0002	−0.015
		Lingual gyrus	−3.67	3854	−26	−71	−4	0.0002	−0.014
		Precentral/postcentral	−3.1	5282	−32	−18	68	0.0002	−0.013
		Insula	−2.21	646	−31	20	−2	0.009	−0.013
	RH LGI	TPJ	−5.05	2413	62	−40	20	0.0002	−0.017
		DMPFC/VMPFC/DLPFC	−4.61	4197	23	56	15	0.0002	−0.015
		Lateral occipital	−3.7	3126	37	−88	−14	0.0002	−0.014

**Fig. 1:**
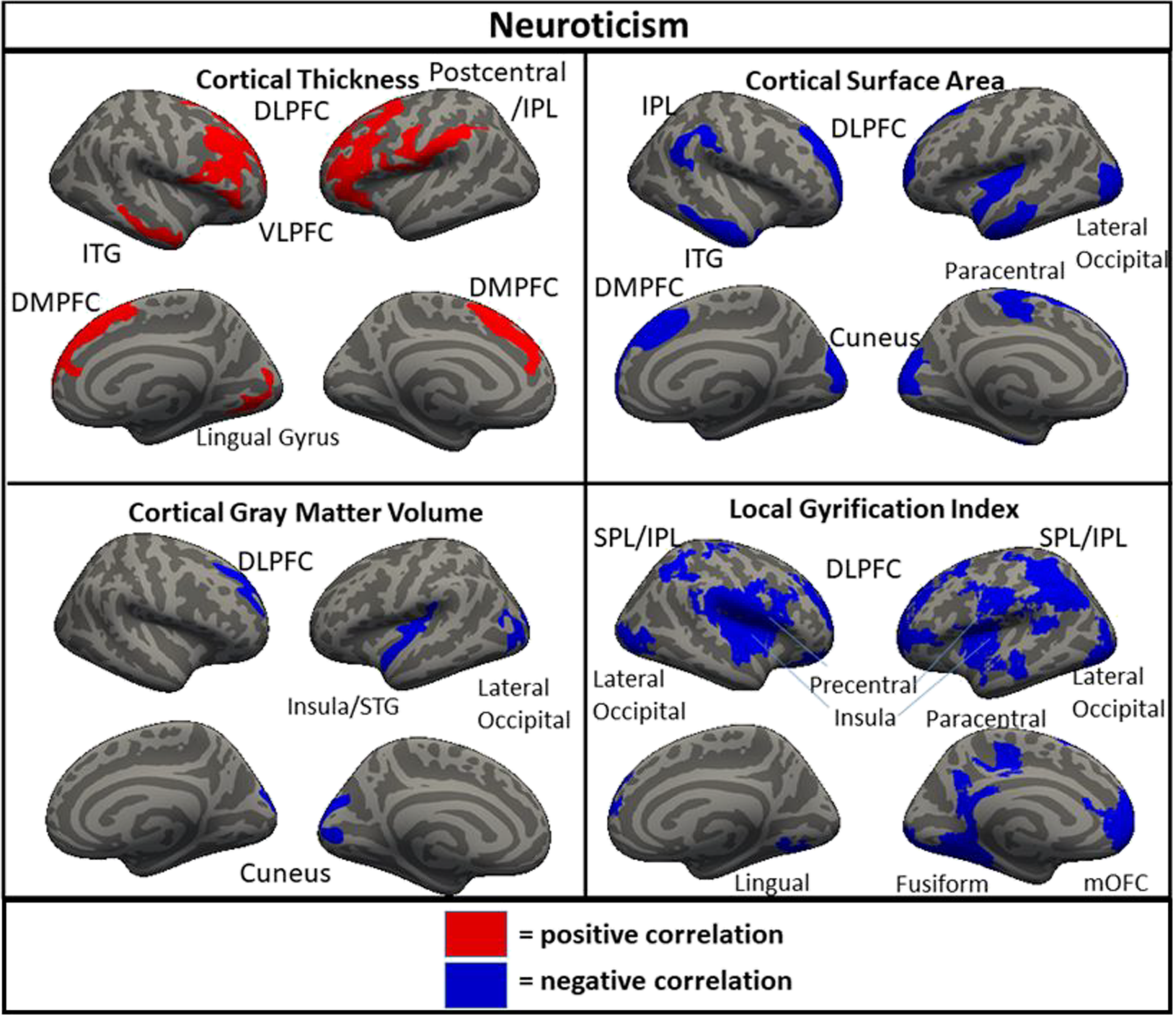
Clusters of significant association between brain metrics and neuroticism in the full sample (*N* = 1104). DLPFC = dorsolateral prefrontal cortex; ITG = inferior temporal gyrus; VLPFC = ventrolateral prefrontal cortex; DMPFC = dorsomedial prefrontal cortex; STG = superior temporal gyrus; IPL = inferior parietal lobule; SPL = superior parietal lobule; mOFC = medial orbitofrontal cortex.

**Table 3:** Brain by personality correlations across the original, replication and full samples. Cluster’s from each sample organized based on hemisphere, lobe and lateral/medial surface. Covariates included age, sex, estimated intracranial volume and the other four FFM traits.

**FFM Trait**	**Metric**	**Region**	***N* = 507**	***N* = 597**	***N* = 1104**
**N**	LH thickness	Lateral frontal	X	X	X
		Medial frontal	X	X	X
		Lateral parietal	X		X
	RH thickness	Lateral frontal	X	X	X
		Medial frontal	X		X
		Lateral parietal	X		
		Lateral temporal	X		X
	LH area	Lateral frontal	X	X	X
		Medial frontal		X	
		Medial parietal	X		X
		Lateral temporal	X		X
		Lateral occipital	X	X	X
		Medial occipital	X		X
	RH area	Lateral frontal	X	X	X
		Medial frontal	X	X	X
		Lateral parietal	X		X
		Lateral temporal	X		X
		Medial occipital			X
	LH volume	Lateral temporal	X		X
		Lateral occipital		X	X
		Medial occipital	X		X
	RH volume	Lateral frontal			X
		Medial temporal	X		
	LH LGI	Lateral frontal	X	X	X
		Medial frontal	X	X	X
		Lateral parietal	X	X	X
		Medial parietal	X	X	X
		Lateral temporal	X		X
		Medial temporal		X	X
		Lateral occipital	X		X
		Medial occipital	X		X
	RH LGI	Lateral frontal	X	X	X
		Medial frontal	X		X
		Lateral parietal	X	X	X
		Medial parietal	X		
		Lateral temporal	X	X	X
		Lateral occipital	X		X
		Medial occipital	X		X
**E**	LH thickness	Medial frontal			X
		Lateral parietal			X
		Medial parietal	X		X
		Lateral temporal		X	
	RH area	Lateral temporal	X		
	LH volume	Medial temporal	X		
	RH volume	Lateral frontal			X
		Medial temporal	X		
	LH LGI	Lateral parietal		X	X
	RH LGI	Lateral parietal		X	
		Medial temporal	X		X

**Table 3:** (continued)

**FFM Trait**	**Metric**	**Region**	***N* = 507**	***N* = 597**	***N* = 1104**
**O**	LH thickness	Lateral frontal	X	X	X
		Lateral parietal	X		X
	RH thickness	Lateral frontal	X	X	X
		Medial frontal	X		
		Lateral parietal	X		
		Lateral occipital	X		
	LH area	Lateral temporal	X		X
	RH area	Lateral parietal	X		
		Lateral occipital	X		
	LH volume	Lateral temporal	X		X
	RH volume	Lateral temporal	X		X
	LH LGI	Lateral frontal		X	X
		Medial frontal	X		X
		Lateral parietal		X	
		Medial parietal	X		
		Lateral temporal	X		X
		Lateral occipital	X		X
		Medial occipital	X		
	RH LGI	Medial temporal	X		
		Lateral occipital			X
**A**	LH thickness	Lateral frontal	X		X
	RH thickness	Medial occipital			X
		Medial occipital			X
	RH area	Medial temporal	X		
	RH volume	Lateral frontal	X		X
	LH LGI	Lateral frontal		X	
		Medial frontal		X	
		Medial temporal	X		
		Medial occipital		X	X
	RH LGI	Lateral parietal		X	X
		Medial parietal	X		
		Lateral temporal	X		
		Medial temporal		X	
		Lateral occipital		X	X
**C**	LH thickness	Lateral frontal	X	X	X
	RH thickness	Lateral frontal	X	X	X
		Medial parietal	X		
	LH area	Lateral frontal		X	X
		Lateral temporal	X		X
		Lateral occipital	X		
	RH area	Lateral frontal		X	X
		Medial frontal		X	X
		Lateral temporal	X		
	LH volume	Lateral temporal	X		
		Lateral occipital	X		
	RH volume	Lateral temporal	X		
	LH LGI	Lateral frontal	X	X	X
		Medial frontal	X	X	X
		Lateral parietal	X	X	X
		Lateral temporal	X		X
		Lateral occipital	X		
		Medial occipital			X
	RH LGI	Lateral frontal		X	X
		Medial frontal	X	X	X
		Lateral parietal	X	X	X
		Lateral occipital	X		X

#### Neuroticism

In the full sample for neuroticism, a cluster of positive correlation was found spanning the DLPFC, VLPFC and DMPFC for thickness in both the right and the left hemisphere (Figure 1). Additionally, clusters were found in the left hemisphere only in the postcentral gyrus and the IPL, and clusters were found in the right hemisphere only in the inferior temporal gyrus (ITG) and lingual gyrus. Additionally, clusters of negative correlation were found for neuroticism with area in the left insula/STG, lateral occipital cortex, paracentral gyrus and cuneus, as well as the in the right DLPFC/DMPFC, ITG, IPL and cuneus. For volume, clusters of negative correlation were found in the insula/STG, cuneus and lateral occipital, as well the right cuneus and DLPFC. For LGI, clusters of negative association were found in the left DLPFC (two), fusiform gyrus, SPL/IPL (two), precentral gyrus/insula and paracentral lobule, as well as in the right SPL/IPL, DLPFC, precentral gyrus/insula, medial orbitofrontal cortex, lateral occipital cortex and lingual gyrus. Notably, when the other four FFM traits were not controlled, area in the left DLPFC and DMPFC was associated with neuroticism, but volume in the right DLPFC was not.

#### Extraversion

**Fig. 2:**
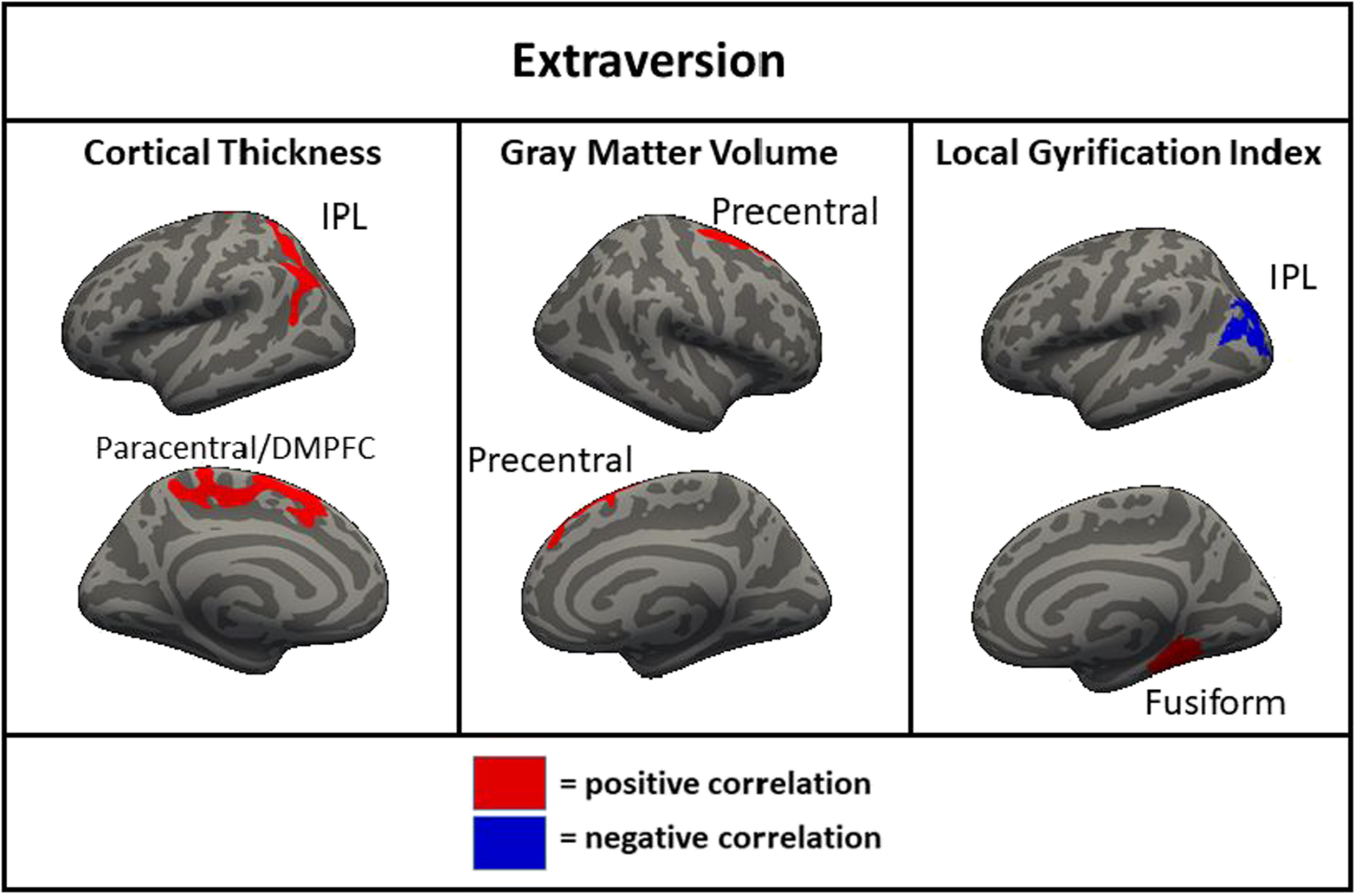
Clusters of significant association between right hemisphere gray matter volume and extraversion in the full sample (*N* = 1104). IPL = inferior parietal lobule.

For extraversion, a cluster of positive association was found for thickness in the left IPL and DMPFC/paracentral lobule and in the right precentral gyrus, as well as a positive cluster of association for volume in the right precentral gyrus (Figure 2). A cluster of positive association with LGI in the left IPL was found, as well as a cluster of negative association with LGI in the fusiform gyrus. Notably, when the other four FFM traits were not controlled, there were no regions for which cortical thickness was associated with extraversion.

**Fig. 3:**
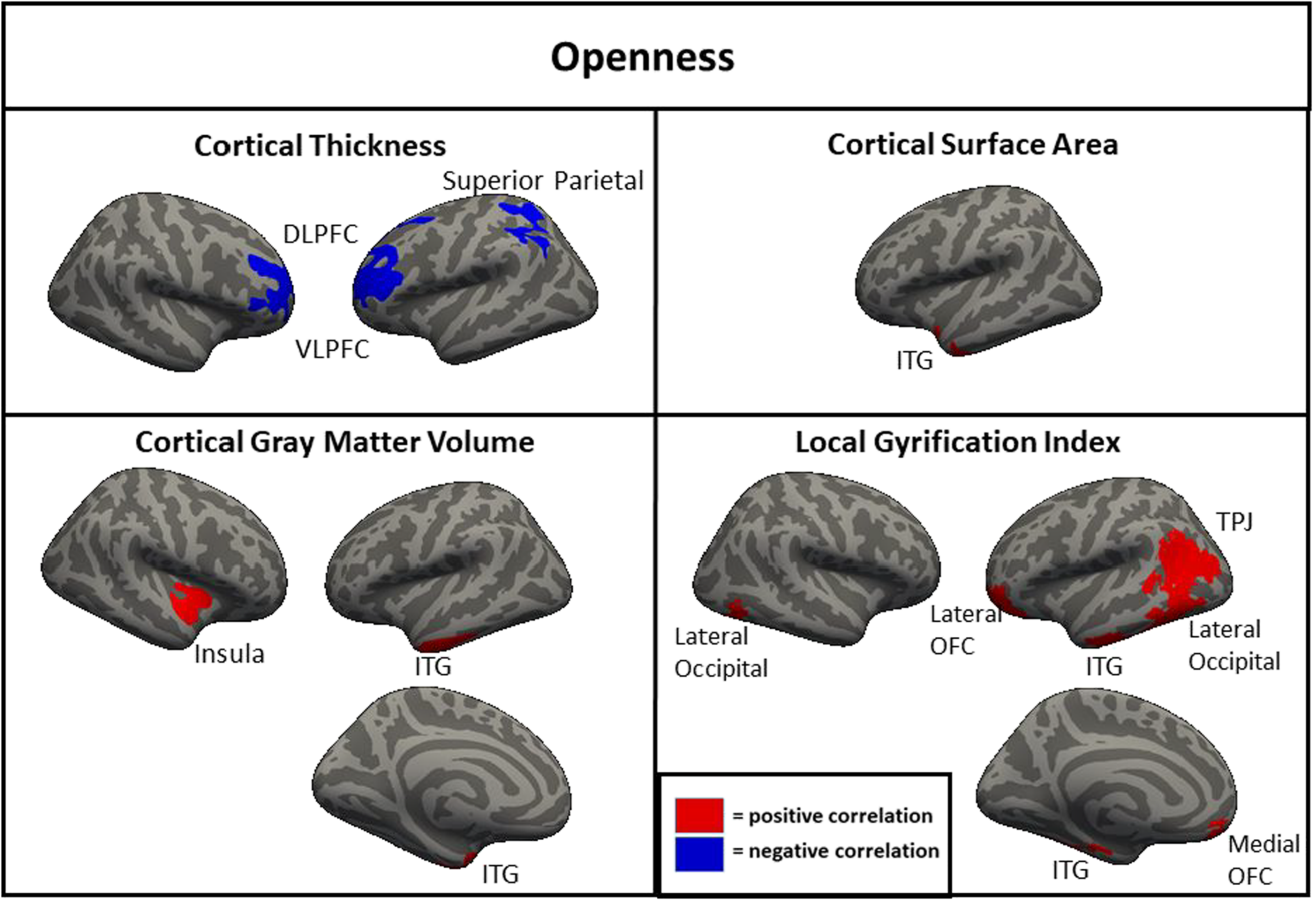
Clusters of significant association between brain metrics and openness in the full sample (*N* = 1104). DLPFC = dorsolateral prefrontal cortex; ITG = inferior temporal gyrus; VLPFC = ventrolateral prefrontal cortex; STG = superior temporal gyrus; IPL = inferior parietal lobule; SPL = superior parietal lobule; OFC = orbitofrontal cortex; TPJ = temporoparietal junction.

#### Openness

For openness, clusters of negative correlation with cortical thickness were found in the left and the right DLPFC/VLPFC and in the left SPL (Figure 3). Clusters of positive association of openness were found with area and volume in the left inferior temporal cortex and for volume in the right insula. Additionally, clusters of positive association were found with LGI in the left lateral/medial orbitofrontal cortex, left inferior temporal cortex, left temporoparietal junction/lateral occipital cortex and right lateral occipital cortex. Notably, when the other four FFM traits were not controlled, LGI in the right lateral and medial orbitofrontal cortex was also associated with openness.

#### Agreeableness

**Fig. 4:**
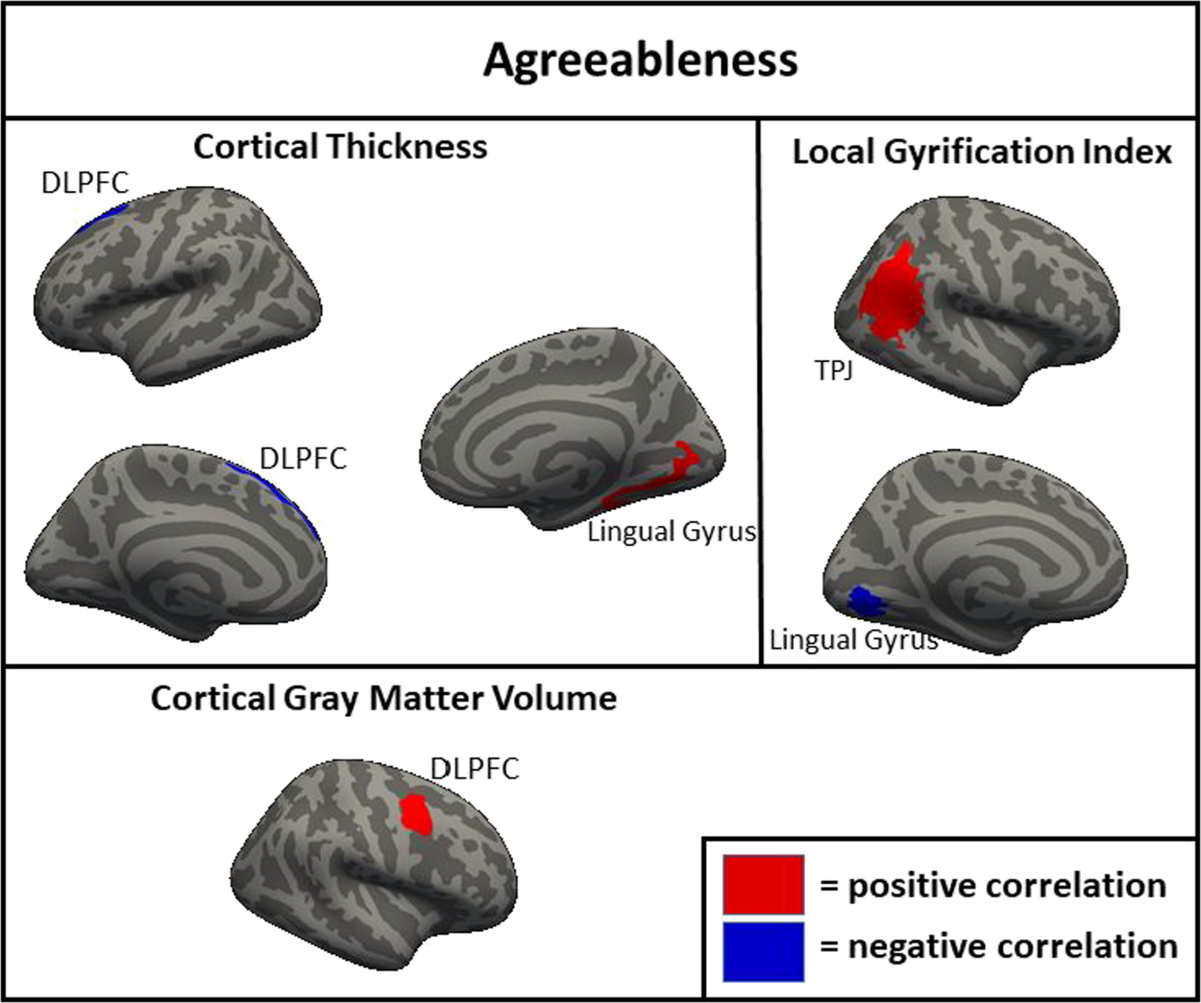
Clusters of significant association between brain metrics and agreeableness in the full sample (*N* = 1104). DLPFC = dorsolateral prefrontal cortex; TPJ = temporoparietal junction.>

Clusters of association with agreeableness were found for thickness in the left DLPFC (negative) and the right lingual gyrus (positive; Figure 4). For the correlation of agreeableness and volume, a cluster of positive association was found in the right DLPFC. For the association of agreeableness and LGI, clusters of negative association were found in the lingual gyrus (negative) and the fusiform gyrus (positive). Notably, when the other four FFM traits were not controlled, surface area in the right DLPFC and LGI in the left DLPFC were also positively associated with agreeableness.

**Fig. 5:**
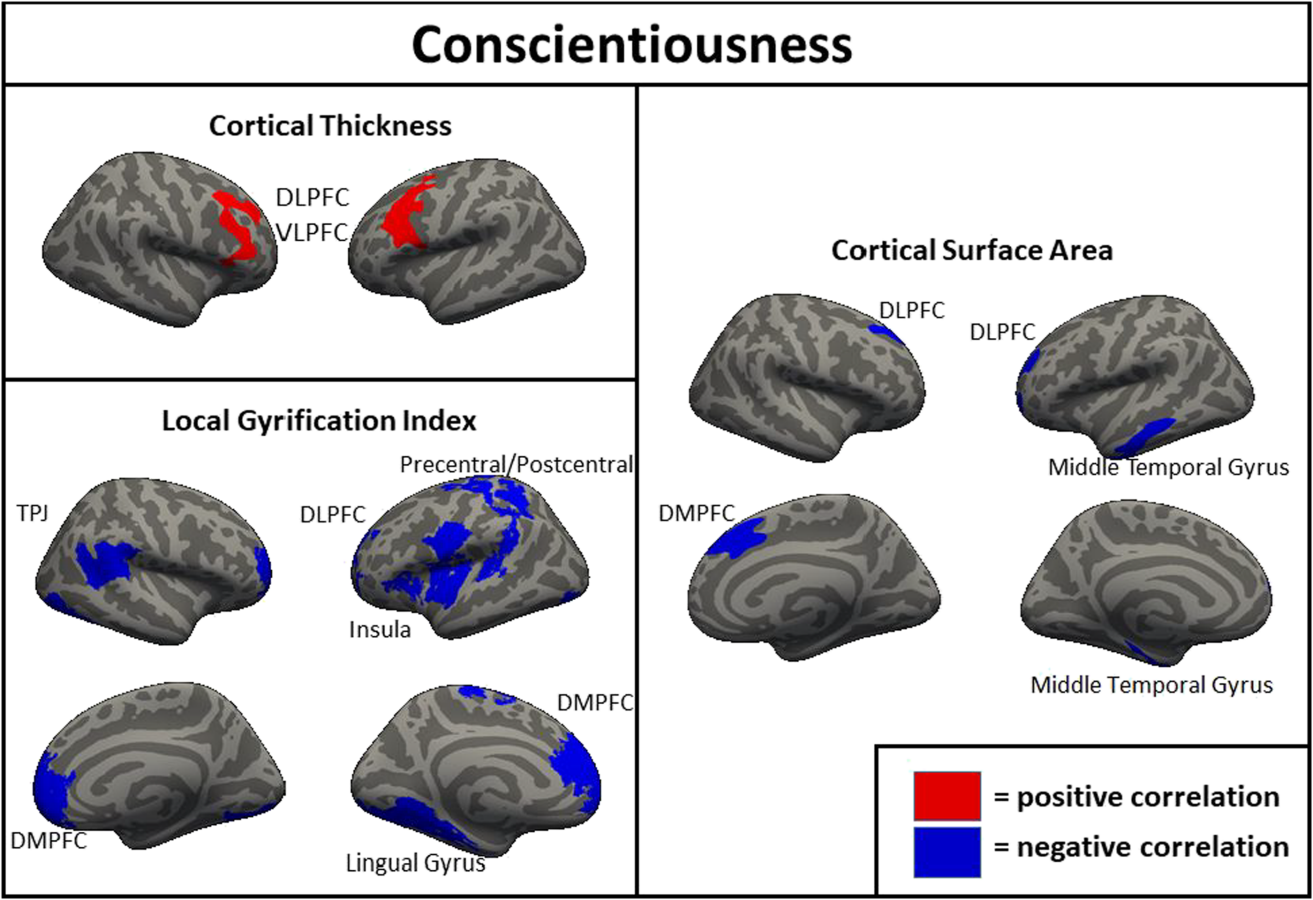
Clusters of significant association between brain metrics and conscientiousness in the full sample (*N* = 1104). DLPFC = dorsolateral prefrontal cortex; VLPFC = ventrolateral prefrontal cortex; DMPFC = dorsomedial prefrontal cortex; TPJ = temporoparietal junction.

#### Conscientiousness

For conscientiousness, clusters of positive association were found for thickness in the right and the left DLPFC/VLPFC (Figure 5). For the association of conscientiousness and area, clusters of negative association were found in the left middle temporal gyrus and DLPFC, as well as the right DLPFC/DMPFC. For the association of conscientiousness and LGI, clusters of negative association were found in the left precentral gyrus, DLPFC/DMPFC, lingual gyrus, precentral/postcentral gyrus and insula, as well as in the right temporoparietal junction, DMPFC/VMPFC/DLPFC and lateral occipital cortex. Notably, when the other FFM traits were not controlled neither thickness nor surface area in the the DLPFC was associated with conscientiousness.

## Discussion

The FFM has been used as a framework for understanding a wide range of important outcomes (Ozer and Benet-Martínez, 2006; Soto, submitted for publication), but research into the neuroanatomical structures underlying FFM traits has been generally limited by inconsistent methodologies and small samples. The current study was designed to replicate and extend the results of Riccelli *et al*. (2017) in an independent sample of comparable size drawn from the same population using the same acquisition methods and then aggregate the results of their original sample and the replication sample. The data from the full sample (*N* = 1104) represent the largest investigation of the structural morphometry of personality to date and contribute to our understanding of how latent constructs like personality traits manifest physiologically.

The neuroanatomical correlates of neuroticism, openness and conscientiousness were generally consistent across the full, original and replication samples. In both subsamples and the full sample, neuroticism was associated with greater cortical thickness in the DLPFC and DMPFC; less surface area of the right DLPFC and DMPFC; and less gyrification of the DLPFC, DMPFC, lateral parietal cortex and right lateral temporal cortex. Openness was associated with less cortical thickness in the left and the right DLPFC in both subsamples and the full sample. Conscientiousness was associated with greater cortical thickness in the left and the right DLPFC and less gyrification in the DLPFC, DMPFC and lateral parietal cortex. These associations found across both subsamples likely represent the most robust findings with the greatest likelihood of replicating in other samples. There were also many associations found in the full sample and one of the two subsamples but not the other (e.g. associations of agreeableness with thickness, area and volume in the DLPFC), as well as associations found only in one subsample (e.g. associations of openness with thickness and area in the lateralty parietal and occipital cortices). Additionally, there were findings which were significant in the full sample only, suggesting a small-sized effect detectable only with a very large sample, including a negative correlation of extraversion and volume in the right DLPFC and negative correlations of conscientiousness with area and volume in the right temporal parietal junction.

One interesting finding that was consistent across the current and previous studies was the inverse relationship between cortical thickness and cortical surface area/LGI. According to one prominent theory, there is a process that occurs during development in which the brain is ‘stretched’ so that the surface area and gyrification of the cortex increases while the cortical ribbon becomes thinner (Hogstrom *et al*., 2013). This is thought to be an indicator of cortical maturation that occurs to improve communication between areas of the brain throughout development (Ruppin *et al*., 1993; Murre and Sturdy, 1995) and is supported by studies showing inverse relationships between cortical thickness and surface area/LGI (Hogstrom *et al*., 2013). The current results were generally consistent with this interpretation, as in almost all cases, the relationship between a trait and cortical thickness was the opposite direction as the relationship between the same trait with cortical surface area and LGI. Additionally, since volume is most strongly associated with surface area, the majority of trait by volume associations was in the same direction as area and LGI. Neuroticism, the FFM trait most linked to internalizing psychopathology (Kotov *et al*., 2010), was associated with less area, LGI and volume, and with greater cortical thickness suggesting that less developmental ‘stretching’ in critical areas may be a contributing factor to this trait and related indices of psychological functioning. In contrast, openness and agreeableness were associated with greater surface area, LGI and volume, and with less cortical thickness suggesting that these traits may be the result of greater stretching of certain areas in development. The differential relationship of these morphometric features to openness is consistent with prior work showing similar patterns of association with intelligence, a trait that is highly related to facets of openness (Schnack *et al*., 2015; Kaufman *et al*., 2016). Less clear is the interpretation of the direction of findings of morphometry with extraversion and conscientiousness. Conscientiousness generally showed directional associations with morphometry that were similar to neuroticism, a somewhat surprising finding given that the two traits were inversely associated (*r* = −0.40). This was less pronounced but still present even when the other FFM traits were not controlled. There were generally few associations of morphometry and extraversion in analyses with either covariate strategy and those that were present were not consistent in direction, with LGI positively and negatively associated with extraversion in different regions, and thickness and volume both positively associated with extraversion.

The large number of regions associated with each trait suggests that neuroanatomical basis of personality is based in many small effects throughout the brain rather than one single region of the brain. This is plausible given the substantial complexity of personality as a latent construct that manifests at multiple levels of analysis (e.g. cognitive, affective, behavioral, motivational, etc.). However, the individual regions whose morphometry was most frequently associated with personality across both subsamples and full sample were DLPFC and DMPFC, which were identified across the traits of neuroticism, openness, and conscientiousness. Additionally, the thickness, area, volume and gyrification of the DLPFC were found to be associated with agreeableness in one subsample and the full sample. This comes despite the fact that all non-focal FFM traits were being controlled, suggesting that the DLPFC and DMPFC likely represent the regions of the brain most associated with personality overall. The DLPFC’s status as one of the strongest morphometric correlate of personality is not surprising, as it is implicated in a diverse array of cognitive abilities (Duncan and Owen, 2000). This region is considered to be integral to the performance of tasks requiring executive functioning (Niendam *et al.*, 2012). Dysfunction of the DLPFC is thought to play a key role in depression (Koenigs and Grafman, 2009), which many empirical approaches to mental illness consider to be underlain by the trait of neuroticism (Kotov *et al.*, 2017). Transcranial magnetic stimulation of the DLPFC has been demonstrated to improve decision making, reduce craving for food and addictive substances and is approved by the Food and Drug Administration for the treatment of depression (Jansen *et al.*, 2013; Li *et al.*, 2013; Berlim *et al.*, 2014; Brunoni *et al.*, 2017; Lowe *et al*., 2017; Yang *et al*., 2018). Thus, the relationship of the structure of the DLPFC with neuroticism, openness and conscientiousness may be the result of one of many functions for which the DLPFC is integral.

However, one consideration regarding these associations comes in regard to conscientiousness, which was no longer associated with thickness in the left or right DLPFC or LGI in the left DLPFC when the other FFM traits were not controlled. Given that the direction of conscientiousness findings was unexpected when controlling for the other FFM traits (i.e. consistent with directional findings of neuroticism and not the other three traits, despite a negative bivariate correlation between these two traits), it is possible that results for conscientiousness in the DLPFC may represent a suppression effect resulting from the removal of variance relating to the other traits. In adolescence and young adulthood, greater cortical thinning in the DLPFC is typically seen as a marker of successful neurodevelopment (Giedd and Rapoport, 2010) and thinner DLPFC is associated with better cognitive abilities (e.g. working memory: Owens *et al.*, 2018), which would seem consistent with higher levels of conscientiousness. On the other hand, there is theoretical reason to think that the structure of the DLPFC would relate to conscientiousness, as it is conceptualized as a trait akin to impulsivity and related to difficulties with response inhibition (Wiggins, 1996; Whiteside and Lynam, 2001), both of which are traits that have been linked to the DLPFC previously. Additionally, previous voxel-based morphometry studies have found gray matter volume in the DLPFC to relate to conscientiousness (DeYoung *et al.*, 2010), though these studies also controlled the other FFM traits. Thus, there is a reason to believe that brain morphometry in the DLPFC may be important to the trait of conscientiousness, but also reasons for caution in interpreting this finding.

These findings highlight that the question of whether to control for the other FFM traits is a meaningful one. The current study completed its full-sample analysis using both approaches and found similar, but not identical results across approaches. We think that *not* controlling for the other FFM traits is the preferable analytic approach, since there are documented difficulties in interpreting the relations of partialed personality variables with criterion variables from their broader nomological network (Lynam *et al.*, 2006; Sleep *et al.*, 2017). The interpretative concerns related to partialing are more serious when two variables are highly interrelated (i.e. partialing the variance of variable X out of variable Y when X and Y have a large bivariate relation). However, in the current full sample, neuroticism and conscientiousness are correlated at *r* = −0.40 (16% shared variance), which is meaningful when considering effect sizes are as small as those identified in the current results. Moreover, the differences between the two approaches, particularly in morphometric correlates of conscientiousness, highlight the need for consideration by the field of personality neuroscience regarding which approach is more appropriate and robust. Future research should seek to more fully address the question of statistically controlling for other traits when conducting studies in personality neuroscience. Until a field-wide consensus for best practice guidelines is reached on this issue, we advocate that researchers justify their use of covariates and to consider pre-registration as a strategy for inoculating themselves from criticisms about selective reporting (Hyatt *et al*., under review).

## Considerations and conclusions

Some findings were present in Riccelli *et al.* (2017) and the current study while others were not. Replicability between the two samples was relatively good for findings relating to neuroticism and conscientiousness. For neuroticism, the thickness, area and LGI of the DLPFC and DMPFC were found in both studies, as well as area in the lateral occipital cortex and LGI specifically in the lateral parietal cortex and lateral temporal cortex. For conscientiousness, both studies identified associations with thickness of the DLPFC and LGI in the DLPFC, DMPFC and lateral parietal cortex. For openness, the association with thickness of the DLPFC (found in both hemispheres) replicated. For agreeableness and extraversion, no regional metrics were associated in both studies, though several were found in one study and the full sample. In summary, these findings suggest that neuroticism, conscientiousness and openness may be the FFM traits that have the most robust neuroanatomical representation. They also suggest that individual differences in neuroanatomy in the lateral and medial frontal, lateral parietal, lateral temporal and lateral occipital cortices may be the regions most associated with individual differences in personality traits.

Another important consideration of the current work relates to the sample and the generalizability of these findings given that the current sample comprised relatively young, healthy adults from the United States. Similar efforts are needed in more diverse (in terms of age, race, ethnicity) samples, as recent studies on the morphometric correlates of complex behaviors have found differing neural correlates between adolescents and adults. For example, two large sample studies of the morphometric correlates of delayed reward discounting found substantially different regions underpinning delayed reward discounting when studied in adults (Owens *et al.*, 2017) and adolescents (Mackey *et al.*, 2017). While there were also differences in methodology (SBM *vs* voxel-based morphometry), this incongruity suggests the possibility of significant differences between adults and adolescents in morphometric correlates of complex behaviors.

The current results suggest that there are numerous cortical structures that are relevant to personality, and the structure of the DLPFC appears to play a role in the neuroanatomical underpinnings of several personality traits. In addition to investigating the morphometric correlates of personality traits, the current study contributes to our understanding of the replicability of personality neuroscience findings broadly. Despite an increasing awareness of the problems of replicability in social science (Ioannidis, 2005; Simmons *et al.*, 2011; Button *et al.*, 2013; Miguel *et al.*, 2014), many seminal studies have not been reanalyzed or replicated. The current study is one of a small number of instances to attempt to replicate a large initial study in an equally large sample. Despite the strengths of the study by Riccelli *et al*. (2017), including an unusually large sample size for a neuroimaging study and state-of-the-art data acquisition and analysis methods, many regional associations identified in their sample were not found in an independent sample of similar size drawn from the same population using the exact same methods. Furthermore, there were some regions found in the original sample did not replicate in the aggregated analysis including both subsamples. Based on the high quality of the original study (i.e. advanced methodology, large sample size), this outcome likely represents the more optimistic end of the spectrum of replicability. In other words, it is likely that attempts to replicate other morphometric correlation studies that used smaller and demographically different samples would detect even fewer replicable findings (e.g. Gray *et al.*, 2018). The current findings suggest regions whose structure is most related to personality, but more importantly they accentuate the importance of statistical power and replication to the future of personality neuroscience research.

## Funding

This material is based upon work conducted by Dr. Nathan T Carter who is supported by the National Science Foundation under grant SES-1561070. No funding sources were involved in study design or collection, analysis, and interpretation of the data. These findings do not reflect the official position of the National Institutes of Health.

## Supplementary Material

scan-18-117-File002_nsz017
