# Corrigendum to: Cortical morphometry of the five-factor model of personality: findings from the Human Connectome Project full sample

**DOI:** 10.1093/scan/nsab036

**Published:** 2021-03-31

**Authors:** Max M Owens, Courtland S Hyatt, Joshua C Gray, Nathan T Carter, James MacKillop, Joshua D Miller, Lawrence H Sweet

**Affiliations:** Department of Psychology, University of Georgia, Athens, GA 30602, USA; Department of Psychology, University of Georgia, Athens, GA 30602, USA; Department of Medical and Clinical Psychology, Uniformed Services University, Bethesda, MD 20814, USA; Department of Psychology, University of Georgia, Athens, GA 30602, USA; Peter Boris Centre for Addiction Research, St. Joseph’s Healthcare Hamilton/McMaster University, Hamilton, ON L8P 3R2, Canada; Department of Psychology, University of Georgia, Athens, GA 30602, USA; Department of Psychology, University of Georgia, Athens, GA 30602, USA

On page 384 of the originally published version of this manuscript, the sentence

“This creates an effect size metric ranging from −1 to 1 with 1 and −1 denoting perfect association with no noise (i.e. perfect positive and negative association) and 0 denoting no relationship considering the error present”

should have read

“This creates an unstandardized effect size metric ranging from −∞ to ∞ with values farther from 0 denoting larger effect sizes (i.e., greater contrast relative to noise) and 0 denoting no relationship considering the error present.”

These details have been corrected only in this corrigendum to preserve the published version of record.

