# Supplementary material for: Cortical morphometry of the five-factor model of personality: findings from the Human Connectome Project full sample
Source: Soc Cogn Affect Neurosci. 2019 Mar 8;14(4):381–95. doi: 10.1093/scan/nsz017 (PMC6523439; doi:10.1093/scan/nsz017)
Supplement: scan-18-117-File002_nsz017 [file scan-18-117-file002_nsz017.docx]

Supplemental Materials 1

Contrast to Noise Ratio

In regression analyses^[[1]](#footnote-1)^, the outcome variable matrix, $\mathbf{Y}$ (an *n* x 1 matrix of outcome variables where *n* is the number of subjects and 1 is the number of *Y* variables), is considered as a function of predictor variables, $\mathbf{X}$ (a *n* x *k*+1 matrix, where *k* is the number of predictor variables, with an additional column containing 1’s; also known as the design matrix), and regression weights, $\mathbf{B}$ (a *k*+1 x 1 matrix of regression coefficients including one intercept and *k* regression slopes). This linear function can be written as:

$\mathbf{Y=XB+E}$ , (1)

where $\mathbf{E}$ is a *n* x 1 matrix of residuals, or prediction error, determined by the difference between observations and predictions about $\mathbf{Y}$, i.e., $\mathbf{E}\boldsymbol{=}\mathbf{Y -}\hat{\mathbf{Y}}$. Within the Freesurfer context it is common to utilize a contrast matrix, $\mathbf{C}$, to test hypotheses regarding regression coefficients. The $\mathbf{C}$ matrix is a 1 x *k+*1 matrix containing 1’s and 0’s that signal whether the corresponding regression coefficient is of interest in the “contrast.” The selected contrast is signified by the $\boldsymbol{\Gamma}$ matrix, where $\boldsymbol{\Gamma=CB}$. The $\boldsymbol{\Gamma}$ matrix is then used to test whether the $\boldsymbol{\Gamma}$ matrix is different than a matrix containing only 0’s, i.e., $H_{0}: \boldsymbol{\Gamma=CB=0}$ using a *t-*test. For example, in a model with two predictors we would have the following $\mathbf{B}$ matrix:

$\mathbf{B=}\left[ \begin{aligned} \beta_{0} \\ \beta_{1} \\ \beta_{2} \end{aligned} \right]$ , (2)

where $\beta_{0}$ is the intercept, or constant, and $\beta_{1}$ and $\beta_{2}$ are slopes corresponding to the predictiors $x_{1}$ and $x_{2}$, respectively. If we were to focus on the value of a single regression slope, say $\beta_{1}$, we would specify our contrast matrix as:

$\mathbf{C=[}\begin{matrix} 0 & 1 & 0 \end{matrix}\boldsymbol{]}$ , (2)

and therefore $\boldsymbol{\Gamma}\boldsymbol{=}\mathbf{CB=}\beta_{1}$, whereas specifying $\mathbf{C=[}\begin{matrix} 0 & 1 & -1 \end{matrix}\boldsymbol{]}$ would result in $\boldsymbol{\Gamma}\boldsymbol{=}\mathbf{CB=(}\beta_{1}-\beta_{2}$), thus comparing the magnitudes of these two regression coefficients. Here – and in most cases – we will only be interested in the case where $\mathbf{C}$ matrix has only a single 1 (i.e., focusing on a single regression slope), with all other elements of $\mathbf{C}$ equal to 0.

In their 2017 study, Riccelli et al. they utilize the contrast-to-noise ratio (CNR) as an effect size measure. This measure is defined as $\frac{\boldsymbol{\Gamma}}{\sigma_{\mathbf{Y -}\hat{\mathbf{Y}}}}\boldsymbol{=}\frac{\mathbf{CB}}{\sigma_{\mathbf{Y -}\hat{\mathbf{Y}}}}$, which in the case of the single predictor simplifies to $\frac{\beta_{k}}{\sigma_{\mathbf{Y -}\hat{\mathbf{Y}}}}$, where $\beta_{k}$ is the regression slope of interest and $\sigma_{\mathbf{Y -}\hat{\mathbf{Y}}}$ is the standard deviation of the regression model residuals. In our search of the literature, we could find no clear use or interpretational guidance on the CNR as an effect size metric. Essentially, the measure is similar to other effect size measures, such as Cohen’s *d*, in that it is making a comparison of an effect against a measure of variability. Whereas a measure such as Cohen’s *d* utilize the *total* variability as a reference point, the CNR utilizes the standard deviation of the model residuals. Thus, the CNR can be interpreted as the size of the unstandardized relation of *x* and *y* relative to the average size of prediction error (unstandardized) of the overall regression model. Thus, this effect size measure only affords comparison across two different regression models if: a) the regression models include the same predictor and outcome variables; and b) those predictor and outcome variables are on identical scales of measurement between regression models. Therefore, in the current case we are able to compare our own CNRs to those of Riccelli et al. (2017).

Two notable features of the CNR are worth noting here. First, the size of the CNR is dependent on the variance unexplained in the model (i.e., the residual standard deviation), such that the better the overall model is, the higher the CNR will be. Thus, the addition of other predictors – including control variables – will make the effects appear larger despite the actual effect remaining unchanged. Second, although Riccelli et al. (2017) suggest their use CNR aimed to “…provide an estimate of effect sizes comparable across personality traits…” (p. 674), this is only true to the extent that the scale of the personality scores for different traits can be considered as being on the same scale (i.e., that a one-point increase in openness scores is equivalent in metric to a one-point increase in Agreeableness), which is likely untenable if personality scores are unstandardized (and is questionable even if they are standardized). However, for our own purposes (to compare our effect sizes to those of Riccelli et al., 2017), they are valid due to our replication having used the exact same personality measures, morphometric measures, and control variables.

Supplemental Table 1

Significant clusters of brain by FFMT trait correlation from replication sample (N = 597) using other four FFM traits as covariates. Age, sex, and total intracranial volume were modeled as covariates. Max = maximum *p­*-value in cluster, -log10 transformed; Size = cluster size in mm^2^; X, Y, Z coordinates in MNI space; CWP = clusterwise probability; ES = effect size. DLPFC = dorsolateral prefrontal cortex; DMPFC = dorsomedial prefrontal cortex; VLPFC = ventrolateral prefrontal cortex; TPJ = temporoparietal junction; IPL = inferior parietal lobule; SPL = superior parietal lobule.

| **Trait** | **Metric** | **Region** | **Max** | **Size** | **X** | **Y** | **Z** | **CWP** | **ES** |
| --- | --- | --- | --- | --- | --- | --- | --- | --- | --- |
| **N** | LH Thickness | DMPFC | 3.33 | 1357 | -9 | 36 | 36 | 0.002 | 0.016 |
|  |  | VLPFC | 3.07 | 1726 | -51 | 9 | 5 | <0.001 | 0.016 |
|  | RH Thickness | DLFPC | 4.31 | 2350 | 54 | 6 | 3 | <0.001 | 0.017 |
|  | LH Area | DLPFC/DMPFC | -4.49 | 1410 | -11 | 50 | 36 | 0.042 | -0.018 |
|  |  | Lateral Occipital | -2.58 | 3883 | -42 | -76 | 11 | <0.001 | -0.015 |
|  | RH Area | DLPFC/DMPFC | -3.98 | 2243 | 7 | 32 | 53 | 0.001 | -0.017 |
|  | LH Volume | Lateral Occipital | -3.09 | 1262 | -39 | -78 | 9 | 0.005 | -0.015 |
|  | LH LGI | VLPFC | -3.23 | 688 | -49 | 32 | 5 | 0.006 | -0.016 |
|  |  | VMPFC | -2.82 | 1283 | -6 | 63 | -7 | <0.001 | -0.015 |
|  |  | Fusiform/ | -2.76 | 2891 | -34 | -74 | -15 | <0.001 | -0.014 |
|  |  | Lingual/PCC |  |  |  |  |  |  |  |
|  |  | IPL | -2.64 | 943 | -39 | -71 | 39 | <0.001 | -0.014 |
|  | RH LGI | TPJ/Insula | -3.11 | 4120 | 50 | -26 | 25 | <0.001 | -0.015 |
|  |  | DLPFC | -2.93 | 1388 | 19 | 40 | 37 | <0.001 | -0.015 |
|  |  | VLPFC/Lateral Orbitofrontal | -2.66 | 568 | 38 | 36 | -8 | 0.028 | -0.015 |
|  |  | Precentral Gyrus | -2.27 | 651 | 43 | 3 | 28 | 0.01 | -0.014 |
| **E** | LH Thickness | Inferior Temporal Gyrus | 3.09 | 1133 | -51 | -22 | -32 | 0.011 | 0.020 |
|  | LH LGI | IPL/Lateral Occipital | -3.21 | 3750 | -36 | -86 | 11 | <0.001 | -0.019 |
|  | RH LGI | IPL | -3.4 | 2143 | 40 | -74 | 28 | <0.001 | -0.019 |
| **O** | LH Thickness | DLPFC/VLPFC | -3.56 | 1745 | -16 | 58 | 15 | <0.001 | -0.017 |
|  | RH Thickness | DLPFC/VLPFC | -2.69 | 1631 | 20 | 60 | 12 | 0.001 | -0.016 |
|  | LH LGI | VLFPC/Lateral Orbitofrontal | 4.62 | 1451 | -40 | 49 | -8 | <0.001 | 0.019 |
|  |  | TPJ | 2.57 | 654 | -37 | -59 | 19 | 0.008 | 0.016 |
| **A** | LH LGI | DLPFC | 3.67 | 805 | -43 | 16 | 41 | 0.002 | 0.020 |
|  |  | Precentral/Postcentral | 3.31 | 2233 | -38 | -24 | 45 | <0.001 | 0.020 |
|  |  | DMPFC | 2.62 | 707 | -15 | 33 | 49 | 0.005 | 0.018 |
|  |  | Lingual Gyrus | -2.38 | 710 | -10 | -78 | -4 | 0.005 | -0.018 |
|  | RH LGI | TPJ/IPL/SPL/ | 4.71 | 6445 | 46 | -62 | 35 | 0 | 0.020 |
|  |  | Postcentral/Lateral Occipital |  |  |  |  |  |  |  |
|  |  | Parahippocampal Gyrus | -2 | 628 | 34 | -33 | -15 | 0.013 | -0.018 |
| **C** | LH Thickness | DLPFC/VLPFC | 4.26 | 2704 | -45 | 27 | 31 | 0 | 0.020 |
|  | RH Thickness | DLPFC | 2.76 | 1305 | 27 | 33 | 34 | 0.004 | 0.018 |
|  | LH Area | DLPFC | -2.79 | 1648 | -12 | 64 | 12 | 0.015 | -0.018 |
|  | RH Area | DLPFC/DMPFC | -3.03 | 2112 | 7 | 31 | 52 | 0.002 | -0.019 |
|  | LH LGI | DLPFC/DMPFC | -3.59 | 2408 | -22 | 47 | 32 | <0.001 | -0.018 |
|  |  | Precentral/Postcentral | -3.46 | 1388 | -51 | -5 | 27 | <0.001 | -0.019 |
|  |  | Postcentral/SPL | -3.14 | 2379 | -23 | -36 | 60 | <0.001 | -0.018 |
|  | RH LGI | TPJ | -4.4 | 1562 | 60 | -38 | 25 | <0.001 | -0.020 |
|  |  | DLPFC/DMPFC | -3.51 | 3264 | 23 | 56 | 15 | <0.001 | -0.019 |
|  |  | SPL | -2.72 | 938 | 26 | -42 | 55 | <0.001 | -0.018 |
|  |  | Precentral/DLPFC | -2.69 | 684 | 43 | 1 | 41 | 0.006 | -0.018 |

Supplemental Table 2

Significant clusters of brain by FFMT trait correlation from replication sample (N = 1104) **without** using other four FFM traits as covariates. Age, sex, and total intracranial volume were also modeled as covariates. Max = maximum *p­*-value in cluster, -log10 transformed; Size = cluster size in mm^2^; X, Y, Z coordinates in MNI space; CWP = clusterwise probability; ES = effect size. DLPFC = dorsolateral prefrontal cortex; DMPFC = dorsomedial prefrontal cortex; VLPFC = ventrolateral prefrontal cortex; TPJ = temporoparietal junction; IPL = inferior parietal lobule; SPL = superior parietal lobule.

| Trait | Metric | Region | Max | Size | X | Y | Z | CWP | ES |
| --- | --- | --- | --- | --- | --- | --- | --- | --- | --- |
| N | LH Thickness | DLPFC/VLPFC/  DMPFC/Precentral | 5.33 | 7846 | -11 | 30 | 51 | <0.001 | 0.011 |
|  |  | Precentral/Postcentral/  Inferior Parietal | 3.72 | 1728 | -56 | -2 | 8 | <0.001 | 0.011 |
|  | RH Thickness | DLPFC/DMPFC | 4.53 | 2631 | -36 | 14 | 33 | <0.001 | 0.012 |
|  |  | DLPFC | 3.83 | 1210 | -24 | 38 | 33 | 0.007 | 0.011 |
|  | LH Area | DLPFC | -4.32 | 2922 | -10 | 25 | 57 | <0.001 | -0.011 |
|  |  | Cuneus | -3.33 | 2730 | -4 | -85 | 13 | <0.001 | -0.010 |
|  |  | Inferior Temporal | -2.81 | 1436 | -44 | -13 | -37 | 0.023 | -0.010 |
|  | RH Area | DLPFC/DMPFC | -4.87 | 4500 | -37 | 24 | 22 | <0.001 | -0.012 |
|  |  | Cuneus | -2.87 | 1453 | -40 | -75 | 21 | 0.035 | -0.010 |
|  | LH Volume | Cuneus | -2.97 | 2079 | -11 | -85 | 1 | <0.001 | -0.011 |
|  | LH LGI | VLPFC | -5.22 | 1366 | -46 | 34 | 7 | <0.001 | -0.011 |
|  |  | DLPFC | -4.86 | 2493 | -29 | 9 | 53 | <0.001 | -0.011 |
|  |  | Fusiform/Lingual/Lateral Occipital/PCC | -3.56 | 4946 | -36 | -52 | -18 | <0.001 | -0.010 |
|  |  | IPL/TPJ | -3.21 | 4899 | -49 | -53 | 37 | <0.001 | -0.010 |
|  |  | Postcentral Gyrus | -2.70 | 684 | -58 | -16 | 34 | 0.007 | -0.010 |
|  |  | VMPFC | -2.28 | 964 | -11 | 49 | 8 | <0.001 | -0.009 |
|  | RH LGI | DLPFC/VLPFC/Insula/Superior Temporal | -4.61 | 8734 | 61 | -4 | 11 | <0.001 | -0.011 |
|  |  | IPL/SPL/Postcentral Gyrus | -3.65 | 5108 | 31 | -30 | 67 | <0.001 | -0.010 |
|  |  | Lateral Occipital Cortex | -3.21 | 897 | 31 | -88 | 0 | <0.001 | -0.010 |
| E | RH Volume | Precentral Gyrus | 3.26 | 1184 | -49 | -2 | 34 | 0.008 | 0.013 |
|  | LH LGI | DLPFC | 2.78 | 506 | -20 | 26 | 45 | 0.050 | 0.012 |
|  | RH LGI | Fusiform Gyrus | 2.40 | 1204 | 33 | -46 | -19 | <0.001 | 0.011 |
| O | LH Thickness | DLPFC/VLPFC/  DMPFC/Precentral | -4.88 | 7275 | -15 | 57 | 17 | <0.001 | -0.013 |
|  |  | Superior Parietal Lobule | -4.70 | 1143 | -17 | -76 | 39 | 0.011 | -0.013 |
|  |  | Inferior Parietal Lobule | -3.03 | 1005 | -48 | -60 | 41 | 0.024 | -0.012 |
|  | RH Thickness | DLPFC/VLPFC  /DMPFC | -4.08 | 4615 | -37 | 35 | 29 | <0.001 | -0.012 |
|  | LH Area | Inferior Temporal | 2.97 | 1421 | -49 | -11 | -36 | 0.025 | 0.013 |
|  | RH Area | Lateral Occipital | 3.24 | 1438 | -23 | -87 | -12 | 0.036 | 0.012 |
|  | LH Volume | Inferior Temporal | 2.76 | 919 | -36 | -4 | -42 | 0.044 | 0.012 |
|  | RH Volume | Lateral Occipital | 3.56 | 1057 | -18 | -91 | -8 | 0.018 | 0.012 |
|  | LH LGI | Lateral/Medial Orbitofrontal | 5.13 | 2783 | -40 | 49 | -8 | <0.001 | 0.013 |
|  |  | TPJ | 4.15 | 6411 | -52 | -47 | -20 | <0.001 | 0.013 |
|  |  | Inferior Temporal/Parahippocampal Gyrus | 3.53 | 738 | -48 | -16 | -36 | 0.003 | 0.013 |
|  | RH LGI | Parahippocampal Gyrus | 3.04 | 2336 | 47 | -68 | -12 | <0.001 | 0.011 |
|  |  | Lateral/Medial Orbitofrontal | 2.34 | 1512 | 20 | 45 | -12 | <0.001 | 0.011 |
| A | LH Thickness | DLPFC/precentral | -4.50 | 1481 | -38 | 20 | 45 | 0.001 | -0.014 |
|  |  | DLPFC/precentral | -4.34 | 2860 | -21 | 23 | 56 | <0.001 | -0.014 |
|  | RH area | DLPFC/precentral | 4.25 | 1567 | -20 | 4 | 54 | 0.022 | 0.015 |
|  | RH Volume | DLPFC/precentral | 3.00 | 1089 | -19 | 12 | 55 | 0.014 | 0.014 |
|  | LH LGI | Lingual Gyrus | -2.69 | 626 | -11.5 | -83 | -13 | 0.011 | -0.012 |
|  |  | DLFPC | 2.56 | 740 | -36 | 3 | 35 | 0.003 | 0.012 |
|  | RH LGI | Inferior Parietal | 3.94 | 7094 | 54 | -53 | 35 | <0.001 | 0.014 |
| C | RH Area | TPJ | -3.02 | 1688 | -6 | -87 | 32 | 0.014 | -0.012 |
|  | RH Volume | TPJ | -4.03 | 985 | -6 | -86 | 28 | 0.028 | -0.014 |
|  | LH LGI | Precentral Gyrus | -3.24 | 556 | -51 | -5 | 26 | 0.026 | -0.013 |
|  |  | DMPFC | -2.53 | 1563 | -8 | 53 | 36 | <0.001 | -0.012 |
|  | RH LGI | TPJ | -3.21 | 613 | 61 | -38 | 24 | 0.016 | -0.013 |
|  |  | Lateral Occipital | -3.21 | 1498 | 42 | -77 | -13 | <0.001 | -0.012 |
|  |  | DMPFC/DLPFC | -2.99 | 2170 | 13 | 65 | 2 | <0.001 | -0.012 |

Supplemental Table 3

Significant clusters of brain by FFM trait correlation from the full sample (N = 1104) using other four FFM traits as covariates **with an initial cluster forming threshold of *p* < .001**. Age, sex, and total intracranial volume were also modeled as covariates. Max = maximum *p­*-value in cluster, -log10 transformed; Size = cluster size in mm^2^; X, Y, Z coordinates in MNI space; CWP = clusterwise probability; ES = effect size. DLPFC = dorsolateral prefrontal cortex; DMPFC = dorsomedial prefrontal cortex; VLPFC = ventrolateral prefrontal cortex; TPJ = temporal parietal junction.

| **Trait** | **Metric** | **Region** | **Max** | **Size** | **X** | **Y** | **Z** | **CWP** | **ES** |
| --- | --- | --- | --- | --- | --- | --- | --- | --- | --- |
| **N** | LH Thickness | Caudal Middle Frontal Gyrus | 5.1 | 427 | -36 | 11 | 54 | 0.0002 | 0.018 |
|  |  | Superior Frontal Gyrus | 4.8 | 476 | -13 | 31 | 49 | 0.0002 | 0.018 |
|  |  | Superior Frontal Gyrus | 4.8 | 214 | -8 | 28 | 39 | 0.02 | 0.018 |
|  |  | Supramarginal Gyrus | 3.9 | 199 | -58 | -25 | 24 | 0.03 | 0.017 |
|  | RH Thickness | Superior Frontal Gyrus | 5.8 | 832 | 15 | 39 | 42 | 0.0002 | 0.019 |
|  |  | Rostral Middle Frontal Gyrus | 4.8 | 199 | 19 | 54 | 22 | 0.03 | 0.018 |
|  |  | Rostral Middle Frontal Gyrus | 4.7 | 378 | 39 | 33 | 31 | 0.0004 | 0.018 |
|  | LH Area | Inferior Temporal Gyrus | -4.8 | 1014 | -41 | -8 | -38 | 0.0002 | -0.018 |
|  |  | Cuneus | -3.7 | 279 | -4 | -88 | 12 | 0.03 | -0.017 |
|  |  | Superior Frontal Gyrus | -3.6 | 582 | -18 | 61 | 9 | 0.0002 | -0.017 |
|  | RH Area | Superior Frontal Gyrus | -5.4 | 1885 | 9 | 47 | 43 | 0.0002 | -0.019 |
|  | LH Volume | Superior Temporal | -4.9 | 192 | -40 | -26 | -2 | 0.03 | -0.018 |
|  | LH LGI | Caudal Middle Frontal Gyrus | -4.9 | 137 | -28 | 9 | 52 | 0.01 | -0.018 |
|  |  | Fusiform Gyrus | -4.7 | 1648 | -28 | -57 | -16 | 0.0002 | -0.018 |
|  |  | Rostral Middle Frontal Gyrus | -4.1 | 126 | -37 | 52 | 4 | 0.02 | -0.017 |
|  |  | Superior Parietal Lobule | -3.5 | 113 | -30 | -48 | 35 | 0.03 | -0.017 |
|  | RH LGI | Supramarginal Gyrus | -4.9 | 327 | 54 | -24 | 23 | 0.0002 | -0.018 |
|  |  | Precentral Gyrus | -4.7 | 175 | 60 | -3 | 12 | 0.003 | -0.018 |
|  |  | Insula | -3.7 | 163 | 37 | -16 | -6 | 0.004 | -0.016 |
| **E** | LH Thickness | Inferior Parietal Lobule | 4.3 | 228 | -35 | -73 | 45 | 0.02 | 0.021 |
| **O** | LH Thickness | Rostral Middle Frontal Gyrus | -3.7 | 194 | -32 | 48 | 8 | 0.03 | -0.017 |
|  | RH Volume | Superior Temporal Gyrus | 4.4 | 234 | 43 | -13 | -13 | 0.01 | 0.019 |
|  | LH LGI | Lateral Orbitofrontal Cortex | 4.6 | 133 | -22 | 43 | -13 | 0.01 | 0.018 |
| **A** | RH Area | Caudal Middle Frontal Gyrus | 4.7 | 368 | 39 | 3 | 50 | 0.01 | 0.022 |
| **C** | LH Thickness | Rostral Middle Frontal Gyrus | 4.3 | 271 | -45 | 26 | 30 | 0.006 | 0.021 |
|  | LH LGI | Superior Frontal Gyrus | -3.9 | 110 | -16 | 58 | 14 | 0.04 | -0.020 |
|  | RH LGI | Supramarginal Gyrus | -5.0 | 469 | 62 | -40 | 20 | 0.0002 | -0.022 |
|  |  | Superior Frontal Gyrus | -4.2 | 105 | 14 | 64 | 3 | 0.04 | -0.021 |

1. To simplify this discussion, we only consider the subject-level analyses, and thus ignore vertex-level weights matrix. [↑](#footnote-ref-1)
